# Efficacy of Radiomics in Predicting Oncologic Outcome of Liver-Directed Combined Radiotherapy in Locally Advanced Hepatocellular Carcinoma

**DOI:** 10.3390/cancers15225405

**Published:** 2023-11-14

**Authors:** Jong Won Park, Hansang Lee, Helen Hong, Jinsil Seong

**Affiliations:** 1 Department of Radiation Oncology, Yonsei Cancer Center, Yonsei University College of Medicine, 50-1 Yonsei-ro, Seodaemun-gu, Seoul 03722, Republic of Korea; jongwon987@yuhs.ac; 2 School of Electrical Engineering, Korea Advanced Institute of Science and Technology, 291 Daehak-ro, Yuseong-gu, Daejeon 34141, Republic of Korea; hansanglee@kaist.ac.kr; 3 Department of Software Convergence, College of Interdisciplinary Studies for Emerging Industries, 621 Hwarang-ro, Nowon-gu, Seoul 01797, Republic of Korea

**Keywords:** hepatocellular carcinoma, liver-directed combined radiotherapy, radiomics, treatment response, in-field failure-free survival rate

## Abstract

**Simple Summary:**

In hepatocellular carcinoma (HCC), the clinical predictive factors for tumor markers are well-known. Although these factors are recognized as essential, recent attempts have been made to predict treatment outcomes using radiomics based on imaging markers. We investigated whether radiomic features extracted from three-phase dynamic contrast-enhanced computed tomography (CECT) can be used to predict clinical outcomes, including objective treatment response (OR) and in-field failure-free survival rate (IFFR), in 409 patients with HCC who received liver-directed combined radiotherapy (LD-CRT). In predicting the OR and IFFR, clinical models and radiomics models based on tumoral and peritumoral areas showed an acceptable performance, while combined clinico-radiomics models (CCR) performed better. Therefore, CCR models have potential use in clinical prediction. Moreover, the constructed nomograms based on these models may provide valuable information on the OR and IFFR in patients with HCC undergoing LD-CRT.

**Abstract:**

Purpose: We investigated whether radiomic features extracted from three-phase dynamic contrast-enhanced computed tomography (CECT) can be used to predict clinical outcomes, including objective treatment response (OR) and in-field failure-free survival rate (IFFR), in patients with hepatocellular carcinoma (HCC) who received liver-directed combined radiotherapy (LD-CRT). Methods: We included 409 patients, and they were randomly divided into training (n = 307) and validation (n = 102) cohorts. For radiomics models, we extracted 116 radiomic features from the region of interest on the CECT images. Significant clinical prognostic factors are identified to predict the OR and IFFR in the clinical models. We developed clinical models, radiomics models, and a combination of both features (CCR model). Results: Among the radiomic models evaluated for OR, the OR-PVP-Peri-1cm model showed favorable predictive performance with an area under the curve (AUC) of 0.647. The clinical model showed an AUC of 0.729, whereas the CCR model showed better performance (AUC 0.759). For the IFFR, the IFFR-PVP-Peri-1cm model showed an AUC of 0.673, clinical model showed 0.687, and the CCR model showed 0.736. We also developed and validated a prognostic nomogram based on CCR models. Conclusion: In predicting the OR and IFFR in patients with HCC undergoing LD-CRT, CCR models performed better than clinical and radiomics models. Moreover, the constructed nomograms based on these models may provide valuable information on the prognosis of these patients.

## 1. Introduction

Hepatocellular carcinoma (HCC) is the most common primary cancer of the liver and the fourth leading cause of cancer-related deaths worldwide [1]. Although treatment modalities have developed and reached a certain degree, the prognosis of HCC remains poor owing to tumor recurrence, and the 5 year overall survival is around 10–20% even after curative treatment options [2,3,4,5] (surgical resection, ablation, or liver transplantation).

For the treatment of advanced HCC, systemic therapy has long been the preferred option, which usually involves sorafenib and, more recently, atezolizumab plus bevacizumab [6,7]. However, in cases of locally advanced HCC, liver-directed combined radiotherapy (LD-CRT) should receive more attention because of its effectiveness, which may enable curative resection [8,9,10]. LD-CRT effectively reduces the size of locally advanced HCC that is initially unsuitable for surgery, leading to improved patient survival rates [9,10]. Additionally, recent studies have shown that selected patients treated with LD-CRT can convert tumors beyond the Milan criteria to those within the Milan criteria, indicating the potential for conversion therapy to curative surgery [8].

Predicting the treatment response is clinically important for cancer treatment. In HCC, the clinical predictive factors for tumor markers are well-known [11,12,13,14]. Although these factors are recognized as essential, recent attempts have been made to predict treatment outcomes using radiomics based on imaging markers. Radiomics has emerged as a new approach to extracting quantitative radiological data from medical images (radiomic data). This involves extracting complex information about the tumor and surrounding tissue characteristics, such as density, texture, shape, borders, and blood flow, to understand the nature of the tumor and explore its correlation with clinical outcomes, such as survival, therapeutic response, and pathology. By building appropriate models with advanced features, radiomic analysis has already proven to be helpful in various types of cancer diagnosis and prognostic prediction and is expected to become increasingly crucial in predicting cancer treatment outcomes in the future, particularly in the fields of radiology and oncology.

We aimed to investigate whether radiomic features extracted from contrast-enhanced dynamic liver computed tomography (CT) scans can correlate with prognostic factors and predict clinical outcomes such as objective response (OR) and in-field failure-free survival rate (IFFR) in patients with HCC undergoing LD-CRT. The predictive accuracy of the model was assessed using an independent validation group. To the best of our knowledge, this study is the first and largest to evaluate prognostic factors and clinical outcomes in patients with HCC undergoing LD-CRT to develop a clinico-radiomics model.

## 2. Materials and Methods

### 2.1. Patients

This retrospective study was conducted by searching electronic medical records. We identified 409 patients with inoperable HCC who underwent LD-CRT between November 2005 and December 2018. The inclusion criteria were as follows: (1) HCC patients who had received LD-CRT; (2) pre-radiation contrast-enhanced three-phase CT performed within two months before radiotherapy; (3) Child–Pugh class A or B disease; and (4) Eastern Cooperative Oncology Group (ECOG) performance status of no more than 2. We excluded patients meeting the following criteria: (1) presence of distant metastasis at the beginning of radiotherapy, (2) previous or concurrent other malignancies, (3) history of radiation to the abdominal area, and (4) incomplete radiotherapy (biologically effective dose [BED] < 40 Gy) owing to patient refusal or poor general condition. The entire cohort was randomly divided into training and validation datasets in a ratio of 7:3. The training dataset was used to construct the models evaluated using the validation dataset. Baseline clinicopathological data, including age, sex, Eastern Cooperative Oncology Group (ECOG) performance status, Child–Pugh score, HCC etiology (hepatitis B, hepatitis C, or neither), diagnosis date, serum alpha-fetoprotein (AFP), serum protein-induced vitamin K absence or antagonist-II (PIVKA-II), indocyanine green (ICG) R15, tumor size, clinical stage, portal vein tumor thrombosis (PVTT), radiation dose, and treatment volume, were obtained from medical records.

Patients were consistently followed up every three months after radiotherapy based on AFP, PIVKA-II, and imaging examinations, and the time of disease-specific progression (in-field failure, out-field failure, nodal failure, or distant metastasis) or death was recorded. Abdominal three-phase contrast-enhanced CT (CECT) was performed every three months. Treatment response was evaluated using the modified response evaluation criteria in solid tumors group (mRECIST) at the three-month visit after completing radiotherapy. Complete response (CR) and partial response (PR) were considered objective responses (OR), whereas stable disease (SD) and progressive disease (PD) were considered non-ORs.

### 2.2. Treatment Protocols

Five-mm margins around the gross tumor volume (GTV) and clinical target volume (CTV) were defined as the CTV and planning target volume (PTV), respectively. Prior to 2010, tumor movement was included in the PTV by adding a generous margin in the craniocaudal direction. Four-dimensional computed-tomography-based planning was adopted in 2010, and the internal target volume (ITV) was delineated considering the tumor movement for every respiratory phase. Additional 5 mm margins around the ITV and CTV were defined as the CTV and PTV, respectively.

The radiotherapy doses were customized to maximize the dose delivered to the tumor while satisfying normal organ dose constraints. For three-dimensional conformal radiotherapy, 45 Gy in 25 fractions is typically prescribed for the PTV. As IMRT was implemented in more patients, our practice pattern shifted towards delivering higher doses of radiation. The GTV or ITV received a radiation dose of 50–75 Gy in 20–25 fractions using the central simultaneous integrated boost (SIB) technique, whereas the surrounding PTV received a lower radiation dose of 45–60 Gy in 20–25 fractions. The GTV minus 1 cm was treated with an SIB of 100 Gy in 25 fractions for selected tumors with sufficient distance from the luminal organs. For equal comparisons of dose effects of various fractionations, the maximum prescribed dose to the tumor was calculated as BED (α/β = 10).

In cases with multiple tumors, the primary and adjacent tumors were irradiated, and lesions outside the target volume were treated with transarterial chemoembolization (TACE) at the time of arterial port insertion. If portal vein tumor thrombosis or regional nodal metastases were present, they were treated in the radiotherapy field.

Continuous hepatic arterial infusion chemotherapy with 5-fluorouracil (500 mg/m^2^/day) during the first and last weeks of radiotherapy was administered using a percutaneous hepatic arterial catheter inserted via hepatic arterial angiography. At 1 month after radiotherapy, hepatic arterial infusion chemotherapy using 5-fluorouracil (500 mg/m^2^ on days 1–3) and cisplatin (60 mg/m^2^ on day 2) was administered every 4 weeks for 1–14 cycles in accordance with the treatment response after radiotherapy and liver function.

### 2.3. CT Scan Protocols

Three-phase CECT was performed at our institute with one of the following machines: a 64-detector row (Aquilion CXL, Toshiba Medical System, Tokyo, Japan) or a 320-detector row CT machine (Aquilion One, Toshiba Medical System, Tokyo, Japan). The same scanning parameters were used for both machines: tube voltage, 120 kV; tube current, 250 mA; and slice thickness, 3 mm, and Br40d for the kernel. All images were reconstructed using filtered back projection (FBP) algorithms. After a routine unenhanced scan, 1.5 mL/kg of contrast medium was injected into the antecubital vein at a rate of 3.0 mL/s via a pump injector. Hepatic arterial phase CT images were obtained at 20–25 s, and portal venous phase CT images were obtained at 35–40 s after injection.

### 2.4. Radiomics Feature Extraction

**Figure 1:**
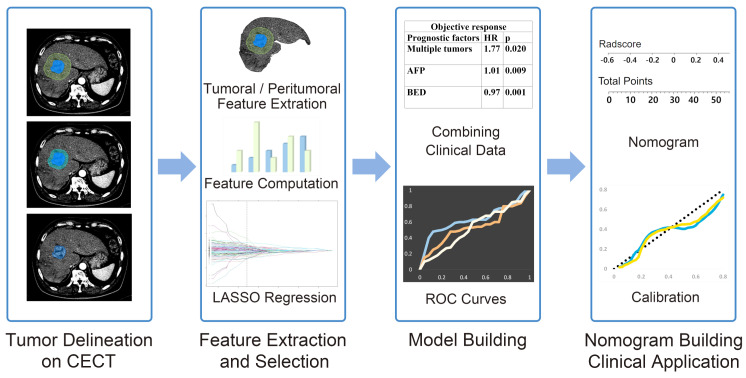
Workflow of radiomics analysis. The radiomics workflow started with three−dimensional segmentation of tumor in three−phase CECT images. After segmentation, handcrafted radiomic features including shape, intensity, and texture were extracted. Least absolute shrinkage and selection operator (LASSO) were used for the radiomic feature selection and model building. Combining radiomics model with clinical features, we obtained the CCR model. Nomogram building and calibration was performed.

The workflow of radiomics analysis are depicted in Figure 1. A radiation oncology expert performed three-dimensional segmentation of the HCC using MIM Software Version 6.5.8 (MIM Software Inc., Cleveland, OH, USA). Regions of interest (ROI) were manually delineated on 3 mm arterial phase and portal venous phase CT images to encompass the entire tumor (ROI tumor). Based on the initial ROI, ROI were reconstructed at 1 cm and 2 cm from the tumor surface, resulting in the assignment of ROI 1cm and ROI 2cm, respectively.

Radiomics features were extracted from the contour images of each ROI, including ROI tumor, ROI 1cm, and ROI 2cm, using MATLAB. In the feature extraction process, we utilized three 2D slice images from one image volume, which comprised the central slice with the largest cross-section area of the tumor and its adjacent slices. During the hand-crafted feature (HCF) extraction process (including original texture, shape, and peritumoral texture), we included 116 texture features for each ROIs, such as histogram characteristics (such as mean, skewness, kurtosis), histogram percentile intensities, gray level co-occurrence matrices (GLCM) features (such as contrast, entropy), gray level run length matrix (GLRLM) features (such as short and long run emphasis), and local binary pattern (LBP) features. Additionally, we included shape features, such as the area/perimeter ratio and eccentricity. The hand-crafted radiomics features are listed in Table 1.

### 2.5. Feature Selection, Model Building, and Model Evaluation

The least absolute shrinkage and selection operator (LASSO) method was used to select useful predictive features from the ROIs and construct a combined clinico-radiomics (CCR) model using multiscale clinical and radiomic features. The discrimination performance of the model was evaluated using the area under the receiver operating characteristic (ROC) curve (AUC) in the primary training and validation groups, with a value of 1 indicating perfect discrimination and 0.5 representing randomness.

The Hosmer–Lemeshow test was applied to the prediction model. We further built a nomogram for the model to provide a more direct method to determine the OR and IFFR. A calibration curve was plotted to analyze the prognostic performance of the nomogram on both the training and validation datasets. The “rms” R package was used for Cox proportional hazards regression, nomograms, and calibration curves. By filling in the CheckList for EvaluAtion of Radiomics Research (CLEAR) checklist, we tried to improve the quality, reliability, and, in turn, reproducibility of this study.

### 2.6. Statistical Analysis

Multivariate binary logistic regression was used to identify significant predictive factors of treatment response. For the IFFR, we used the Kaplan–Meier method to calculate the actuarial curves. The Cox proportional hazards model was used for the univariate and multivariate analyses of independent prognostic clinical factors for each survival rate. Variables significantly associated with survival rates on univariate analysis were selected as candidates for multivariate analysis. The candidate clinical variables included age, sex, ECOG performance status, Child–Pugh score, HCC viral etiology (hepatitis B, hepatitis C, or neither), serum AFP, serum PIVKA-II, tumor size, clinical stage, portal vein tumor thrombosis (PVTT), and radiation dose.

We used SPSS ver. 25 (IBM, Armonk, NY, USA) for statistical analyses, and *p*-values < 0.05 were considered statistically significant.

## 3. Results

### 3.1. Clinical Characteristics

The patient characteristics in the training (n = 307) and validation (n = 102) groups are summarized in Table 2. No significant difference is found in median age (*p* = 0.076), gender (*p* = 0.527), viral etiology (*p* = 0.166), Child–Pugh class (*p* = 0.775), serum albumin level (*p* = 0.187), serum bilirubin level (*p* = 0.516), INR (*p* = 0.401), serum AFP level (*p* = 0.441), serum protein induced by vitamin K absence-II (PIVKA-II) level (*p* = 0.566), tumor size (*p* = 0.737), number of tumors (*p* = 0.550), portal vein thrombosis (*p* = 0.096), and surgery after radiotherapy (*p* = 0.872) between the training and validation groups, meaning the two sets are similarly sampled, which justifies their use as training and validation cohorts.

### 3.2. Clinical Outcomes and Prognostic Factors

Treatment response using the mRECIST showed that 126 (30.8%) patients had CR, 187 (45.7%) had PR, 65 (15.9%) had SD, and 31 (7.6%) had PD. OR rates were 76.5%, whereas local control rates were 92.4%. Using binary logistic regression, tumor multiplicity (*p* = 0.020), AFP level (*p* = 0.009), and BED dose (*p* = 0.001) were considered significant for the OR rate (Table 3). Tumor size (*p* = 0.028), tumor multiplicity (*p* = 0.019), and BED (*p* = 0.001) were significant prognostic factors in multivariate Cox regression analysis (Table 3). These prognostic factors in each clinical outcome were used as clinical features to construct the CCR model for each clinical outcome.

### 3.3. Performance of Radiomics and Combined Clinico-Radiomics Models

**Figure 2:**
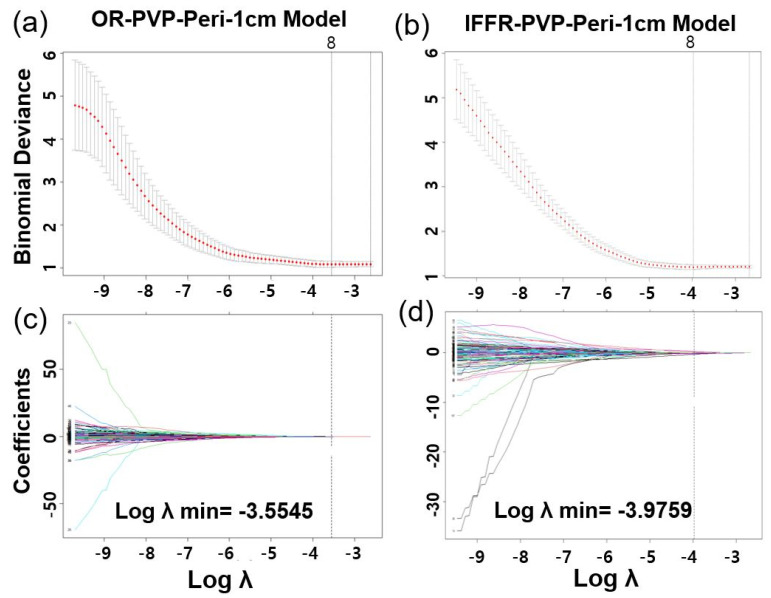
Radiomics feature selection using the LASSO regression. Tuning parameter (λ) selection in the LASSO logistic model for portal venous phase peritumor 1 cm model predicting (**a**) objective response (OR−PVP−Peri−1cm) and (**b**) in−field failure−free survival rate (IFFR−PVP−Peri−1cm). Coefficient profile plots generated by violating the log (λ) sequence for (**c**) OR−PVP−Peri−1cm (8 radiomics features) and (**d**) IFFR−PVP−Peri−1cm (8 radiomics features).

**Figure 3:**
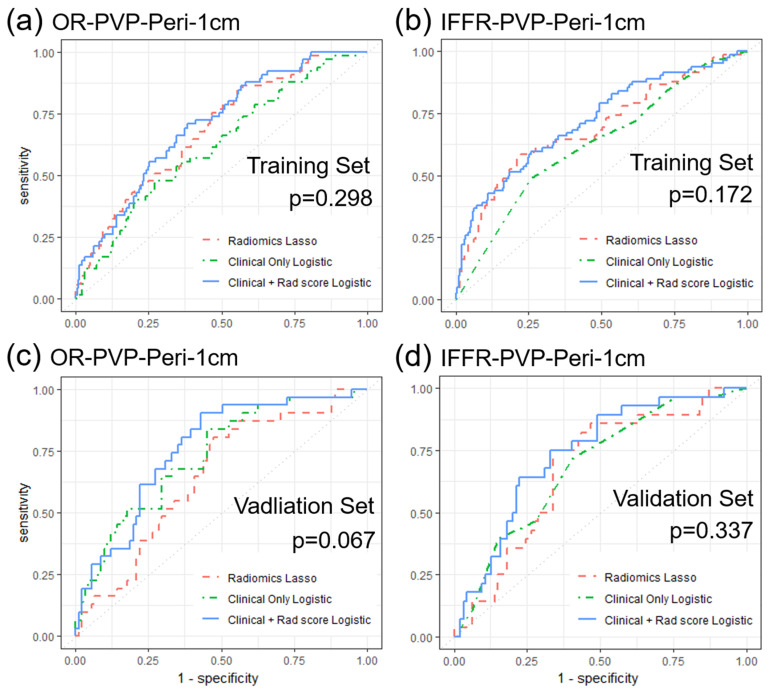
Receiver operating curves (ROC) of the radiomics, clinical, and combined clinico−radiomics model. Portal venous phase peritumor 1 cm model predicting (**a**) objective response (OR−PVP−Peri−1cm) and (**b**) in−field failure−free survival rate (IFFR−PVP−Peri−1cm) in training sets, as well as (**c**) OR−PVP−Peri−1cm, and (**d**) IFFR−PVP−Peri−1cm in validation sets.

The LASSO method was used to select the most useful predictive features from 116 hand-crafted features (HCFs) extracted from the arterial phase (AP) CT or portal venous phase (PVP) CT images of the ROI tumor, ROI 1cm, and ROI 2cm (Figure 2). Among the OR-associated models, the OR-PVP-Peri-1cm model, built using the HCFs in ROI 1cm on portal-venous phase CT, had the largest AUC of 0.647 (95% CI, 0.536–0.749) in the validation set. The OR-PVP-Peri-1cm radiomics model was constructed using eight selected HCFs: entropy, Gray.level.non.uniformity.stdv (GLN.stdv), LBP19, LBP31, Long.run.high.gray.level.emphasis.stdv (LRHGLE.stdv), min, Run.length.non.uniformity.mean (RLN.mean), and Sum.Average.stdv. The clinical model had an AUC value of 0.729 (95% CI, 0.628–0.830) in the validation set, and the combination of the two models (i.e., the CCR model of OR-PVP-Peri-1cm) had a larger AUC of 0.759 (95% CI, 0.665–0.853) than both the radiomic and clinical models. Among the IFFR-associated models, the clinical model had an AUC of 0.687 (95% CI, 0.581–0.793), and the IFFR-PVP-Peri-1cm model had the largest AUC of 0.673 (95% CI, 0.566–0.781) in the validation set. The IFFR-PVP-Peri-1cm model was built using eight selected HCFs: GLN.stdv, Kurtosis, LBP 32, LBP50, LBP 52, LBP9, LRHGLE.mean, and Max. Finally, the CCR model for IFFR-PVP-Peri-1cm had a larger AUC of 0.736 (95% CI, 0.636–0.836) than the clinical and radiomic models. Table 4 shows the AUC of each model with different ROIs, and the ROC curves of the radiomics and CCR models for the objective response and in-field failure-free survival are shown in Figure 3.

### 3.4. Nomogram Construction and Evaluation

**Figure 4:**
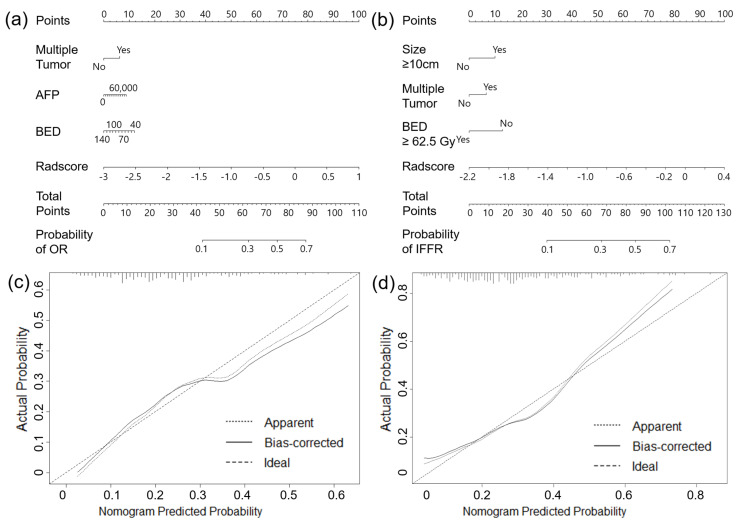
Nomograms and calibration curves for combined clinico−radiomics models. Nomogram for portal venous phase peritumor 1 cm model predicting (**a**) objective response (OR−PVP−Peri −1cm) and (**b**) in−field failure−free survival rate (IFFR−PVP−Peri−1cm). Calibration curves for (**c**) OR-PVP-Peri-1cm and (**d**) IFFR−PVP−Peri−1cm are displayed.

A nomogram was used to provide clinicians with a quantitative tool to predict the individual probabilities of OR and IFFR. As the combined model incorporating the PVP-Peri-1cm radiomics model and clinicopathological factors had the best predictive performance for OR and IFFR, we built a nomogram based on this final model (Figure 4a,b). Calibration curves of the combined nomograms were plotted for the training and validation datasets (Figure 4c,d). The Hosmer–Lemeshow test of the OR-PVP-Peri-1cm and IFFR-PVP-Peri-1cm models show non-significant differences (*p* = 0.322 and *p* = 0.242, respectively) in the validation sets, which demonstrates satisfactory agreement.

## 4. Discussion

In this study, we divided patients with HCC undergoing LD-CRT into training and validation groups. Using three-phase dynamic liver CT, we built radiomic models for both tumoral and peritumoral areas to predict clinical outcomes such as OR and IFFR. The OR-PVP-Peri-1cm and IFFR-PVP-Peri-1cm models show the best performance in predicting the OR and IFFR, respectively. By combining these radiomics models with clinical outcome-predicting prognostic factors obtained from statistical analyses, we developed two CCR models that provide more accurate predictions of clinical outcomes. Two nomograms based on the CCR models were built as a quantitative tool.

With the increasing number of studies on the application of radiomics in HCC, researchers have been progressively investigating the strong predictive capabilities of radiomics. Radiomics, based on various imaging technologies, has broad applications in the diagnosis, treatment, and prognosis of HCC. These include the prognostic prediction, identification, and classification of different HCC types based on disease risk, preoperative diagnosis, treatment response prediction, postoperative recurrence prediction, and many other aspects. Kloth et al. [15] suggested that significant correlations exist between CT texture analysis parameters and those derived from liver perfusion CT computed tomography texture analysis (CTTA). CTTA can aid in the prediction of response and treatment monitoring following DEB-TACE treatment of HCC, complementary to perfusion CT. They also suggested that the correlation between perfusion CT and CTTA parameters may be best in the arterial phase. Park et al. [16] concluded that pre-therapeutic dynamic CT texture analysis can be valuable in predicting complete response (CR) to TACE in patients with HCC, and higher arterial enhancement and gray-level co-occurrence matrix (GLCM) moments, lower homogeneity, and smaller tumor size are significant predictors of CR after TACE. In a study by Zhang et al. [17], texture analysis based on preoperative MRI was a potential quantitative predictor of early recurrence in patients with HCC after hepatectomy. Furthermore, combining the radiomic features of CT and the clinical characteristics of HCC can be used to assess individualized preoperative prediction of OS in patients with HCC portal vein tumor thrombosis undergoing stereotactic body radiotherapy [18]. Several studies have shown the potential utility of a separate peritumoral ROI in the liver parenchyma to improve the diagnostic performance of radiomics for HCC [19]. The radiomics nomogram is a valuable preoperative biomarker that can predict early recurrence of HCC without invasive procedures [20]. Even in patients with small HCC tumors who have undergone surgery or RFA, a radiomic nomogram can be used to predict early recurrence [21]. Survival prediction is another important application in radiomics. Novel deep radiological analysis can be employed to predict the overall survival of patients with HCC undergoing stereotactic body radiotherapy [22]. By combining radiomics features, the radiomics nomogram can deliver a more precise prediction of overall survival compared to the clinicopathological nomogram for patients with HCC following hepatectomy [23].

To construct the radiomics signature, we reduced the 116 candidate radiomics features to a smaller number of potential predictors using the LASSO method. This method considers the predictor–outcome association and shrinks the regression coefficients to select the most relevant factors. It is superior to selecting predictors based solely on their univariate association with the outcome and allows the selected features to be combined into a radiomic signature. However, given the large number of features assessed in radiomics, overfitting poses a considerable risk to the development of radiomic models [24]. To mitigate this risk, a minimum of 10–15 patients per assessed feature is recommended for radiomic studies [25].

In our OR-PVP-Peri-1cm and IFFR-PVP-Peri-1cm models, we selected eight HCFs from the total number of HCFs. For the OR-PVP-Peri-1cm radiomics model, we selected Entropy, GLN.stdv, LBP19, LBP31, LRHGLE.stdv, Min, RLN.mean, and Sum.Average.stdv. For the IFFR-PVP-Peri-1cm radiomics model, we selected GLN.stdv, kurtosis, LBP 32, LBP50, LBP 52, LBP9, LRHGLE.mean, and Max. Entropy specifies the uncertainty/randomness in the image values, measures the average amount of information required to encode the image values, and GLN measures the variability of the gray-level intensity values in the image, with a lower value indicating greater homogeneity in the intensity values. LRHGLE measures the joint distribution of long-run lengths with higher gray-level values, whereas RLN measures the similarity of run lengths throughout the image, with a lower value indicating greater homogeneity among the run lengths in the image. Sum.Average measures the relationship between the occurrence of pairs with lower intensity values and occurrences of pairs with higher intensity values. LBP is a simple grayscale-invariant texture descriptor measure for classification. Max/Min is the maximum/minimum gray level intensity within the ROI, and Kurtosis is a measure of the ‘peakedness’ of the distribution of values in the image ROI. Entropy, GLN, LRHGLE, RLN, and Sum.average are texture features that can be used to describe the spatial variation in intensity within an image and have been used in various applications, such as image segmentation and classification. These features are often calculated using a GLCM, which is a matrix that describes the relationship between the intensity of a pixel and its surrounding pixels. These features are associated with inhomogeneity. The selection of these features implies that radiologic inhomogeneity, which encompasses various aspects of the tumor, such as tumor necrosis, portal vein thrombosis, irregular tumor characteristics and borders, and dilation of the biliary duct by the tumor, may predict the treatment response to radiotherapy and IFFR.

We assessed the relationship between extracted features and clinical outcomes using LASSO regression. Only features with significant diagnostic performance in assessing the prediction target were selected for further analysis. Yuan et al. [26] reported that combining clinicopathological factors with radiomics models resulted in the best predictive power for recurrent-free survival in a validation dataset, with the combined model consisting of portal venous phase radiomics signatures yielding the best results. In our study, combining portal venous phase radiomics with clinical features yielded the best predictive power for OR and IFFR. The AUC of radiomics models, clinical models, and CCR models were 0.647/0.729/0.759 for OR, and 0.673/0.687/0.736 for IFFR, respectively. Combining radiomics features with clinical factors can provide additional information that may improve the accuracy of predicting treatment response or prognosis. While radiomic features can provide information about the tumor’s radiologic properties, clinical factors such as serum AFP level (implying the overall tumor burden) and higher radiation dose (tumor cells are better eradicated) can provide information about the patient’s overall disease status. By integrating these two types of information with CCR model, we can predict outcomes and treatment responses more accurately.

Several studies have shown the potential utility of a separate peritumoral ROI in the liver parenchyma to improve the diagnostic performance of radiomics for HCC [19,27,28]. Shan et al. [19] developed a peritumoral (2 cm) radiomic model in which the prediction accuracy in the validation cohort was fair (AUC 0.80 in the training set vs. 0.79 in the validation set, *p* = 0.47) and significantly improved the accuracy of the preoperative model for predicting early recurrence compared to the tumoral radiomic model. They used a peritumoral ROI delineated with a 2 cm expansion from the lesion, which was based on the current standard for resection margins for HCC. A randomized controlled trial also reported that a margin of 2 cm could decrease the postoperative recurrence rate and improve survival outcomes, indicating that there may be important information within a 2 cm margin [29]. There are available studies [30,31] based on radiomics within the tumoral area. However, these two studies lacked validation based on independent datasets, which may lead to a risk of overfitting the analyses. In our study, the peritumoral radiomic model with a 1 cm margin showed better performance for the OR, and the peritumoral model with a 1 cm margin showed better performance for the IFFR. These results suggest that microscopic disease within a 1 cm margin, which may not be visible, could provide valuable information on tumor response and prognosis.

Despite its potential, the use of radiomics as a clinical biomarker requires further improvements. Clear evidence and greater integration of radiomics and other data are required to confidently accept the role of radiomics in patient management. The prediction of various features using imaging remains challenging, and a more effective evaluation should focus on both the radiomic features of the tumor and its periphery.

This study had several limitations. First, this was a retrospective, single-center study with one radiation oncologist involved in segmentation, which could have introduced bias or affected the analysis. Both inter-observer, and intra-observer agreement were not assessed. Second, there was a class imbalance, with the number of patients in the OR group being much higher than that in the non-OR group (3:1). This could have biased the model towards the majority class (i.e., OR group). Third, we used internal rather than external validity, which makes it difficult to generalize our results to other institutions. Fourth, because liver-directed combined radiotherapy was performed using CT, and tumors were delineated based on CT images at our institution, more information from other imaging devices (e.g., MRI) could not be included in the radiomic evaluation of HCC patients undergoing liver-directed combined RT. Therefore, although this study provides initial evidence that the CCR model can be valuable in predicting OR and IFFR in patients with HCC undergoing liver-directed combined RT, further prospective studies are required to validate these results.

## 5. Conclusions

In conclusion, our findings suggested that radiomic models based on both tumoral and peritumoral areas using pre-radiotherapy three-phase dynamic liver CT in patients with HCC undergoing LD-CRT have favorable predictive performance for OR and IFFR. Furthermore, CCR models were better predictors than radiomic or clinical models alone in predicting treatment outcomes. We constructed radiomic nomograms based on CCR models to predict OR and IFFR, which can potentially aid in clinical decision-making for the pretreatment of HCC patients undergoing liver-directed combined radiotherapy.

## Figures and Tables

**Table 1:** List of hand-crafted radiomics features.

Categories (Number of Features): Features (Feature Numbers)	
**Texture—histogram features (7):** Histogram mean (1), standard deviation (2), minimum (3) and maximum (4) intensities, skewness (5), kurtosis (6), and entropy (7)	**Texture—GLRLM features (22):** Four direction mean and standard deviation of short run emphasis (27,28), long run emphasis (29,30), gray-level non-uniformity (31,32), run length non-uniformity (33,34), run percentage (35,36), low gray-level run emphasis (37,38), high gray-level run emphasis (39,40), short run low gray-level emphasis (41,42), short run high gray-level emphasis (43,44), long run low gray-level emphasis (45,46), long run high gray-level emphasis (47,48)
**Texture—percentile intensities at (5):** 5% (8), 25% (9), 50% (10), 75% (11), 95% (12)	
**Texture—GLCM features (14):** Four direction mean and standard deviation of angular second moment (13,14), contrast (15,16), sum average (17,18), sum variance (19,290), sum entropy (21,22), entropy (23,24), and difference entropy (25,26)	**Texture—LBP features (59):** 10 uniform patterns in LBP histogram (49–107)
	**Shape features (9):** Area/perimeter ratio (108), convex area (109), eccentricity (110), major axis length (111), minor axis length (112), perimeter (113), solidity (114), Min curvature (115), Mean curvature (116)

**Table 2:** Patient and tumor characteristics in training and validation sets.

Characteristics	Training Set (n = 307)	Validation Set (n = 102)	*p*
Age (years)	56 (ranges, 33–83)	60 (ranges, 28–85)	0.076
Sex			
Male	260 (84.7)	89 (87.3)	0.527
Female	47 (15.3)	13 (12.7)	
ECOG PS			
0, 1	293 (95.4)	91 (89.2)	0.133
2	14 (4.6)	11 (10.8)	
Viral etiology			
HBV	254 (82.7)	79 (77.5)	0.166
HCV	19 (6.2)	5 (4.9)	
Non-B, non-C	34 (11.1)	18 (17.6)	
Child–Pugh class			
A	252 (82.1)	85 (83.3)	0.775
B	55 (17.9)	17 (16.7)	
Serum albumin (g/dL)	3.5 (ranges, 2.1–4.8)	3.7 (ranges, 2.0–4.9)	0.187
Serum bilirubin (mg/dL)	0.70 (ranges, 0.20–5.5)	0.70 (ranges, 0.30–4.5)	0.516
INR	1.1 (ranges, 0.80–1.7)	1.1 (ranges, 0.80–1.6)	0.401
AFP (ng/mL)	280 (ranges, 1.70–12,000)	500 (ranges, 1.20–12,000)	0.441
PIVKA-II (mAU/mL)	2000 (ranges, 10–75,000)	1400 (ranges, 11–75,000)	0.566
Tumor size (cm)	9.2 (ranges, 2.0–21)	8.9 (ranges, 2.0–20)	0.737
Number of tumors			
Solitary	161 (52.4)	50 (49.0)	0.550
Multiple	146 (47.6)	52 (51.0)	
PVTT			
Vp0	92 (30.0)	40 (39.3)	0.096
Vp1–2	70 (22.8)	18 (17.6)	
Vp3	81 (26.4)	24 (23.5)	
Vp4	64 (20.8)	20 (19.6)	
Surgery after RT	55 (17.9)	19 (18.6)	0.872

ECOG PS, Eastern Cooperative Oncology Group performance score; HBV, hepatitis B virus; HCV, hepatitis C virus; AFP, alpha-fetoprotein; PIVKA-II, protein induced by vitamin K absence-II; PVTT, portal vein tumor thrombosis; RT, radiation therapy.

**Table 3:** Significant prognostic factors of multivariate analysis on objective response rates and in-field failure-free survival rates.

Objective Response Rates			
Prognostic Factors	HR	95% CI	*p*
Multiple tumors	1.77	1.09–2.86	0.020
AFP	1.01	0.98–1.03	0.009
BED	0.97	0.95–0.99	0.001
**In-field failure-free survival rates**			
**Prognostic factors**	**HR**	**95% CI**	***p***
Tumor size ≥ 10 cm	1.57	1.05–2.36	0.028
Multiple tumors	1.58	1.08–2.31	0.019
BED ≥ 62.5 Gy	0.51	0.35–0.76	0.001

HR, hazard ratio; AFP, alpha-fetoprotein; BED, biologically effective dose.

**Table 4:** Performance of radiomics, clinical, and CCR model on OR and IFFR.

Models	Radiomics Model AUC		Clinical Model AUC		CCR Model AUC	
	Training	Validation	Training	Validation	Training	Validation
Objective Rate						
OR−AP−Tumor	0.500	0.500	0.622	0.729	0.622	0.729
OR−AP−Peri−1cm	0.615	0.614			0.668	0.743
OR−AP−Peri−2cm	0.608	0.600			0.665	0.742
OR−PVP−Tumor	0.748	0.495			0.761	0.710
OR−PVP−Peri−1cm	0.684	0.647			0.704	0.759
OR−PVP−Peri−2cm	0.653	0.610			0.686	0.739
**In-field failure-free survival rate**						
IFFR−AP−Tumor	0.581	0.625	0.626	0.687	0.643	0.659
IFFR−AP−Peri−1cm	0.500	0.500			0.626	0.687
IFFR−AP−Peri−2cm	0.601	0.506			0.666	0.681
IFFR−PVP−Tumor	0.500	0.500			0.626	0.687
IFFR−PVP−Peri−1cm	0.691	0.673			0.718	0.736
IFFR−PVP−Peri−2cm	0.613	0.560			0.671	0.714

## Data Availability

Research data are stored in an institutional repository and will be shared upon request to the corresponding author.
